# Enhanced Surveillance for Coccidioidomycosis, 14 US States, 2016

**DOI:** 10.3201/eid2408.171595

**Published:** 2018-08

**Authors:** Kaitlin Benedict, Malia Ireland, Meghan P. Weinberg, Randon J. Gruninger, Jenna Weigand, Lei Chen, Katharine Perez-Lockett, Catherine Bledsoe, Lynn Denny, Katie Cibulskas, Suzanne Gibbons-Burgener, Anna Kocharian, Emilio DeBess, Tracy K. Miller, Alicia Lepp, Laura Cronquist, Kimberly Warren, Jose Antonio Serrano, Cody Loveland, George Turabelidze, Orion McCotter, Brendan R. Jackson

**Affiliations:** Centers for Disease Control and Prevention, Atlanta, Georgia, USA (K. Benedict, O. McCotter, B.R. Jackson);; Minnesota Department of Health, St. Paul, Minnesota, USA (M. Ireland);; Michigan Department of Health and Human Services, Lansing, Michigan, USA (M.P. Weinberg);; Utah Department of Health, Salt Lake City, Utah, USA (R.J. Gruninger);; Southwest Utah Public Health Department, Cedar City, Utah, USA (J. Weigand);; Washoe County Health District, Reno, Nevada, USA (L. Chen);; New Mexico Department of Health, Las Cruces, New Mexico, USA (K. Perez-Lockett, C. Bledsoe);; Ohio Department of Health, Columbus, Ohio, USA (L. Denny, K. Cibulskas);; Wisconsin Division of Public Health, Madison, Wisconsin, USA (S. Gibbons-Burgener, A. Kocharian);; Public Health Division, Oregon Health Authority, Portland, Oregon, USA (E. DeBess);; North Dakota Department of Health, Bismarck, North Dakota, USA (T.K. Miller, A. Lepp, L. Cronquist);; Pennsylvania Department of Health, Wilkes-Barre, Pennsylvania, USA (K. Warren);; Louisiana Department of Health, Baton Rouge, Louisiana, USA (J.A. Serrano);; Wyoming Department of Health, Cheyenne, Wyoming, USA (C. Loveland);; Missouri Department of Health and Senior Services, St. Louis, Missouri, USA (G. Turabelidze)

**Keywords:** Coccidioides, coccidioidomycosis, epidemiology, endemic diseases, surveillance, valley fever, fungal infections, United States, fungi

## Abstract

Although coccidioidomycosis in Arizona and California has been well-characterized, much remains unknown about its epidemiology in states where it is not highly endemic. We conducted enhanced surveillance in 14 such states in 2016 by identifying cases according to the Council of State and Territorial Epidemiologists case definition and interviewing patients about their demographic characteristics, clinical features, and exposures. Among 186 patients, median time from seeking healthcare to diagnosis was 38 days (range 1–1,654 days); 70% had another condition diagnosed before coccidioidomycosis testing occurred (of whom 83% were prescribed antibacterial medications); 43% were hospitalized; and 29% had culture-positive coccidioidomycosis. Most (83%) patients from nonendemic states had traveled to a coccidioidomycosis-endemic area. Coccidioidomycosis can cause severe disease in residents of non–highly endemic states, a finding consistent with previous studies in Arizona, and less severe cases likely go undiagnosed or unreported. Improved coccidioidomycosis awareness in non–highly endemic areas is needed.

Coccidioidomycosis is a fungal infection caused by inhalation of soil-dwelling *Coccidioides* spp. organisms. Symptomatic infection occurs in ≈40% of cases and usually presents as a self-limiting, influenza-like illness (also called Valley fever) after a 1–3-week incubation period. A small proportion of patients have life-threatening severe pulmonary or disseminated disease (*1*,*2*). In the United States, coccidioidomycosis is known to be endemic in the southwestern states, with hyperendemic foci in Arizona’s Sonoran Desert and California’s southern San Joaquin Valley (*3*). The disease is also endemic in parts of Nevada, New Mexico, Utah, and Texas (*3*) but to a lesser extent. The actual endemic areas are likely broader than previously recognized; for example, *Coccidioides* was found in soil in south central Washington in 2013 and was implicated in locally acquired cases (*4*).

Coccidioidomycosis is reportable in 22 states. To meet the Council of State and Territorial Epidemiologists (CSTE) coccidioidomycosis case definition, cases must fulfill clinical and laboratory criteria (*5*). Approximately 10,000 cases are reported each year through the National Notifiable Diseases Surveillance System (NNDSS), although the number varies markedly by year. NNDSS captures basic demographic information about coccidioidomycosis cases, including the patients’ state and county of residence, age, sex, race, and ethnicity. Some states routinely collect additional information, such as travel history, that is not available in NNDSS. Approximately 65% of cases are reported from Arizona and ≈30% from California (*6*), and the epidemiology and burden of coccidioidomycosis in these states has been well described. Enhanced surveillance in Arizona during 2007–2008 showed substantial disease, including prolonged illness, and quantified the burden on the healthcare system, including an estimated $85 million total hospital charges in 2007 (*7*). In addition, patients who were aware of coccidioidomycosis before seeking healthcare were diagnosed sooner than those who did not know about the disease (*7*), suggesting that community awareness might prevent unnecessary diagnostic workup and antibacterial administration through earlier coccidioidomycosis diagnosis.

Although cases reported from states other than Arizona and California constitute a small proportion of total reported cases (4% in 2015), the number generally increased during the past decade, similar to the overall trend (*6*), indicating that coccidioidomycosis remains a public health problem on a national scale. However, features of cases in non–highly endemic areas have not been systematically described; previous studies of non–outbreak-associated cases are limited to single-state retrospective reviews of existing surveillance data or medical chart data (*8*–*10*). Therefore, we conducted enhanced surveillance in 14 states to better describe the epidemiology, diagnosis, and outcomes of these cases to help inform current routine surveillance practices and guide future awareness and educational efforts in these areas.

## Methods

During January 1–December 31, 2016, routine surveillance conducted in 14 states (Louisiana, Michigan, Minnesota, Missouri, Montana, Nevada, New Mexico, North Dakota, Ohio, Oregon, Pennsylvania, Utah, Wisconsin, and Wyoming) identified coccidioidomycosis cases according to the CSTE case definition. State or local health department personnel contacted patients to participate in a voluntary telephone interview. A parent or guardian was interviewed for patients <18 years of age, and a relative or medical provider could complete the interview if the patient was incapacitated or deceased. Using a standardized questionnaire, health department personnel asked patients about symptoms, healthcare-seeking behaviors, diagnosis, treatment, outcomes, underlying medical conditions, and travel history. They also collected information about laboratory tests used to diagnose coccidioidomycosis from electronic surveillance databases. Some patients who met the CSTE case definition by laboratory criteria and symptoms but who clearly had a different diagnosis (such as histoplasmosis) or whose illnesses were not believed to be clinically consistent with coccidioidomycosis were not contacted for an interview. We further excluded from the analysis interviewed case-patients with compelling evidence that their illness was caused by something other than coccidioidomycosis.

We classified Nevada, New Mexico, and Utah as low-endemic because the risk for coccidioidomycosis is lower in those states than in Arizona and California. The 11 other states in this analysis were not known to be endemic for coccidioidomycosis and were considered nonendemic. We performed descriptive analysis and examined differences between cases in low- versus nonendemic states. We analyzed categorical variables by using χ^2^ or Fisher exact tests and used Wilcoxon rank-sum tests to compare continuous variables. A human subjects review by CDC determined this project to be nonresearch.

## Results

### Interviewed and Noninterviewed Patients

We identified 339 patients who met the CSTE coccidioidomycosis case definition. Of those, 144 (43%) were not interviewed. Forty-five (31%) of those patients were not interviewed because a different illness etiology was identified or the illness was not believed to be clinically consistent with coccidioidomycosis; another 45 (31%) were unable to be contacted, 19 (13%) died, 14 (10%) refused, and no reason was provided for the remaining 21 (15%). Among the 45 noninterviewed patients with a different illness identified or an illness not clinically consistent with coccidioidomycosis, most had histoplasmosis (18 [40%]), 4 (9%) had aspergillosis, and the remainder had other or unspecified diagnoses. In addition, we excluded 9 interviewed patients thought not to have coccidioidomycosis based on laboratory test results and lack of travel to endemic areas, leaving 186 interviewed patients in the final analysis. Therefore, 16% (54/339) of all patients did not have coccidioidomycosis, all but 1 from nonendemic states. Excluding all patients who did not have coccidioidomycosis, the response rate was 65% (186/285). Interviewed and noninterviewed patients were similar in age and sex.

### Demographic Features and Underlying Medical Conditions

Sixty-four (34%) patients were from low-endemic states, and 122 (66%) were from nonendemic states; 109 (59%) patients were male, 89% were white, and the median age was 65 (range 7–91) years (Table 1). Patients in nonendemic states were less likely than those in low-endemic states to be Hispanic or Latino (4% vs. 25%; odds ratio [OR] 0.10, 95% CI 0.03–0.33), were older (median 67 vs. 60 years; p = 0.01), and were more likely to have a yearly household income >$50,000 (61% vs. 41%; p = 0.043). The most common underlying medical conditions were diabetes (19%), heart disease (19%), and cancer (17%). Patients in nonendemic states were more likely to have heart disease than those in low-endemic states (24% vs. 9%; OR 3.0, 95% CI 1.2–7.7). Thirty-six (19%) patients were considered to be immunosuppressed, 61 (34%) reported no underlying conditions, and 12 (7%) reported a previous history of coccidioidomycosis.

**Table 1:** Demographic features and underlying medical conditions of coccidioidomycosis patients in 14 low-endemic and nonendemic US states, 2016*

Characteristic	Value
Total no. patients	186 (100)
Demographics	
Sex	
M	109 (59)
F	77 (41)
Median age, y (range), n = 185	65 (7–91)
Race, n = 170	
White	151 (89)
Black/African American	9 (5)
Asian	1 (0.6)
American Indian or Alaska Native	3 (2)
Other	6 (4)
Hispanic or Latino, n = 175	19 (11)
Health insurance coverage, n = 158	148 (94)
Some college education or higher, n = 137	91 (66)
Annual household income >$50,000, n = 104	56 (54)
Underlying medical conditions	
Smoking, n = 171	
Currently	10 (6)
In the past	74 (43)
None	87 (51)
Asthma requiring an inhaler	20 (11)
COPD or emphysema	18 (10)
Other lung disease	13 (7)
Diabetes	35 (19)
HIV/AIDS	2 (1)
Heart disease	35 (19)
Cancer	32 (17)
Transplant	2 (1)
Liver disease	9 (5)
Kidney disease	9 (5)
Other major illness	49 (26)
No underlying medical conditions reported	61 (34)
Immunosuppressed†	36 (19)
Immunosuppressive medications, n = 165	32 (19)
History of coccidioidomycosis, n = 174	12 (7)

*Values are no. (%) patients except as indicated. n values are provided for categories with <186 responses. †Defined as HIV/AIDS, solid organ or bone marrow transplant, or immunosuppressive medication use.

### Symptoms, Healthcare Use, and Diagnosis

The most common symptoms were cough (65%), fatigue (62%), and shortness of breath (52%) (Table 2). Less than half of patients reported fever (n = 85 [46%]). Patients in nonendemic states were less likely than those in low-endemic states to report chest pain (25% vs. 53%; OR 0.30, 95% CI 0.16–0.57), headache (24% vs. 41%; OR 0.46, 95% CI 0.24–0.87), joint pain (21% vs. 36%; OR 0.48, 95% CI 0.25–0.94), or muscle pain (18% vs. 31%; OR 0.48, 95% CI 0.24–0.98). Patients first sought healthcare a median of 5.5 (range 0–488; interquartile range [IQR] 1–17) days after symptom onset. Most patients first sought care at a primary care office (36%) or emergency department (36%). Seventy percent of patients reported receiving a diagnosis of another illness before being tested for coccidioidomycosis; among those, 63 (55%) said they received a pneumonia diagnosis, and 82 (83%) were prescribed antibacterial medication. Patients in nonendemic states were more likely to have had a chest radiograph performed than those in low-endemic states (94% vs. 73%; OR 5.4, 95% CI 2.1–13.9). More than half of patients (54%) visited a healthcare provider >3 times before being tested for coccidioidomycosis. Patients in nonendemic states were more likely than those in low-endemic states to ask for coccidioidomycosis testing (23% vs. 10%; OR 2.8, 95% CI 1.1–7.2). Most patients were tested by a primary care physician (30%) or a pulmonologist (26%). Median time between seeking healthcare and diagnosis was 38 (range 1–1,654, IQR 16–73) days. Patients in nonendemic states were more likely than those in low-endemic states to have a positive coccidioidomycosis culture (36% vs. 16%; OR 3.0, 95% CI 1.4–6.5) or immunodiffusion test (36% vs. 16%; OR 3.0, 95% CI 1.4–6.5) and less likely to have a positive enzyme immunoassay test (20% vs. 69%; OR 0.12, 95% CI 0.06–0.23) (Table 3).

**Table 2:** Symptoms and healthcare use among coccidioidomycosis patients in 14 low-endemic and nonendemic US states, 2016*

Characteristic	Value
Symptoms	170 (91)
Cough	121 (65)
Fatigue	116 (62)
Shortness of breath	96 (52)
Fever	85 (46)
Night sweats	71 (38)
Chest pain	65 (35)
Chills	60 (32)
Weight loss	60 (32)
Headache	55 (30)
Rash	54 (29)
Joint pain	49 (26)
Muscle pain	42 (23)
Wheezing	38 (20)
Sore throat	35 (19)
Stiff neck	30 (16)
Coughing up blood	13 (7)
Other symptoms	38 (20)
Type of facility where patient first sought care, n = 160	
Emergency room	57 (36)
Primary care	57 (36)
Urgent care	32 (20)
Specialist	9 (6)
Other	5 (3)
Patient first sought care in an endemic state, n = 166†	105 (63)
Patient first sought care in Arizona, n = 166	46 (28)
Ever went to the emergency room, n = 162	91 (56)
No. visits before being tested for coccidioidomycosis, n = 130	
1	33 (25)
2	27 (21)
>2	70 (54)
Type of doctor who first tested for coccidioidomycosis, n = 172	
Primary care	51 (30)
Urgent care	6 (4)
Emergency room	16 (9)
Infectious disease	30 (17)
Pulmonologist	45 (26)
Other	24 (14)
Site of infection, n = 127‡	
Pulmonary	105 (83)
Disseminated	22 (17)
Total no. healthcare visits for coccidioidomycosis, n = 139	
1	28 (20)
2–3	43 (31)
>3	68 (49)
Prescribed antifungal medication, n = 169	115 (68)
Fluconazole	95 (83)
Itraconazole	13 (11)
Voriconazole	4 (4)
Amphotericin B	3 (3)
Median symptom duration, d (range), n = 56	60 (7–1800)
Median symptom duration among patients recovered at interview, d (range), n = 44	38.5 (7–1800)
Median symptom duration among patients not recovered at interview, d (range), n = 12	90 (28–360)
Median time between symptom onset and interview, d (range), n = 107	115 (12–1672)

*Values are no. (%) patients except as indicated. n values are provided for categories with <186 responses. †Arizona, California, Nevada, New Mexico, Texas, Utah, or Washington. ‡Site of infection was defined as pulmonary if lungs were the only body site involved and disseminated if another body part was involved, based on patient self-report.

**Table 3:** Positive laboratory tests for coccidioidomycosis among patients in 14 low-endemic and nonendemic US states, 2016*

Characteristic	Value
Enzyme immunoassay IgM	52 (28)
Enzyme immunoassay IgM only	20 (11)
Enzyme immunoassay IgG	40 (22)
Enzyme immunoassay IgG only	13 (7)
Enzyme immunoassay IgM or IgG	69 (37)
Enzyme immunoassay IgM or IgG only	45 (24)
Immunodiffusion	53 (29)
Immunodiffusion only	18 (10)
Complement fixation	64 (35)
Median highest complement fixation titer, n = 55	8 (2–1024)
Complement fixation only	23 (12)
Complement fixation titer 1:2 only	7 (4)
Histopathology	9 (5)
Histopathology only	1 (0.5)
Culture	53 (29)
Bronchoalveolar lavage	16 (30)
Lung tissue	16 (30)
Sputum	3 (6)
Other body site	12 (23)
Unknown body site	6 (11)
Culture only	26 (14)
Molecular evidence	14 (8)
Culture, histopathology, or molecular evidence	61 (33)

*Values are no. (%) patients except as indicated. n values are provided for categories with <186 responses.

### Treatment and Outcomes

Seventy-seven (43%) patients were hospitalized (median duration 8 [range 3–60] days). Among 115 (68%) patients prescribed antifungal medication, most (95 [83%]) were prescribed fluconazole. Patients in nonendemic states were more likely than those in low-endemic states to be prescribed antifungals (74% vs. 57%; OR 2.1, 95% CI 1.09–4.1). Fifty-four percent of patients were still symptomatic at the time of the interview; the most common symptom that these patients were still experiencing was fatigue (55%). Among patients who had recovered at the time of the interview, median symptom duration was 38.5 (range 7–1,800, IQR 21–90) days. Coccidioidomycosis interfered with 71% of patients’ usual daily activities (median number of days affected 40 [range 2–1,080] days). Among 55 (31%) patients who had a job or were in school, 77% missed work or school (median 19 [range 1–240] days). Four (2%) patients died.

### Travel to Known Endemic Areas

**Figure 1:**
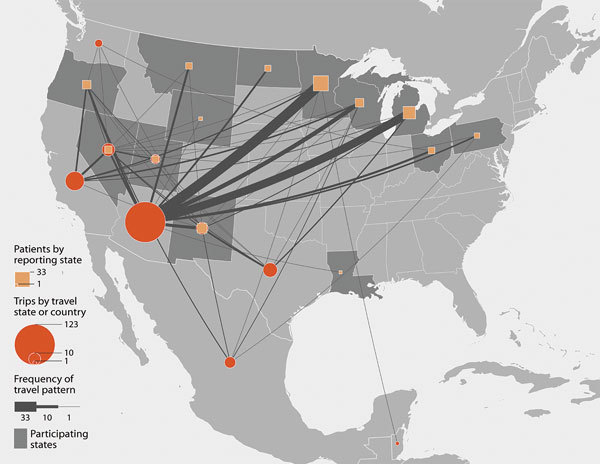
Reporting state and frequency of travel to coccidioidomycosis-endemic areas (Arizona, California, Nevada, New Mexico, Texas, Utah, Washington, Mexico, and Central or South America) in the 4 months before symptom onset or first positive coccidioidomycosis test among coccidioidomycosis patients reported from 14 low-endemic and nonendemic US states, 2016.

**Figure 2:**
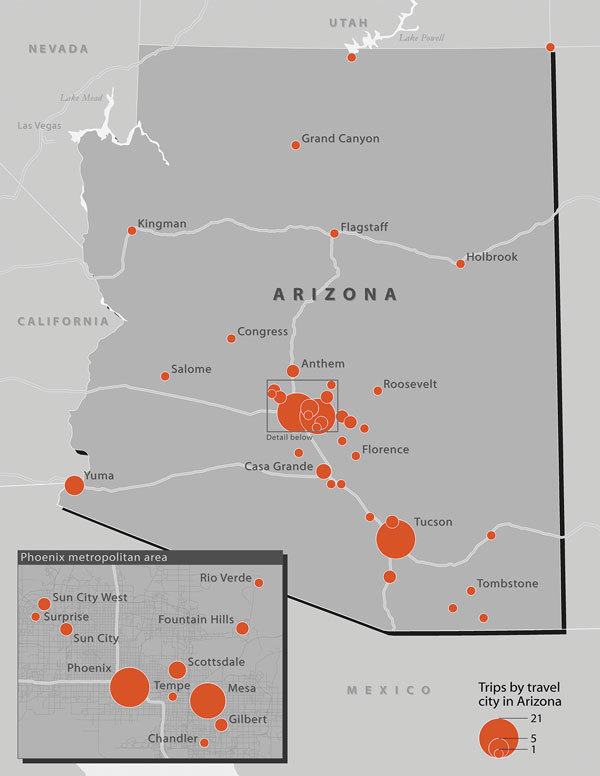
Frequency of trips to Arizona in the 4 months before symptom onset or first positive coccidioidomycosis test among coccidioidomycosis patients reported from 14 low-endemic and nonendemic US states, 2016.

Overall, 124 (68%) patients (26 [41%] from low-endemic states and 98 [83%] from nonendemic states) traveled to Arizona, California, Nevada, New Mexico, Texas, Utah, Washington, Mexico, or Central or South America in the 4 months before symptom onset (or before testing positive, if asymptomatic) (Figures 1, 2). Among 88 patients for whom information was available, median travel duration was 74.5 (range 1–720) days. Five patients, all of whom traveled to Arizona, reported that a travel partner also had coccidioidomycosis. Among patients from nonendemic states, 59 (48%) reported part-time residence in Arizona. Of the 24 patients from nonendemic states who did not travel to known endemic areas in the 4 months before developing coccidioidomycosis, 16 reported ever traveling to those areas in their lifetime; 7 had incomplete travel histories because someone other than the patient was interviewed (n = 5) or because the patient did not complete the interview (n = 2). The remaining patient was a north-central Oregon resident with no underlying medical conditions whose only potentially relevant travel was to Mexico ≈12 years before symptom onset.

### Knowledge of Coccidioidomycosis

Patients in nonendemic states were more likely than those in low-endemic states to know about their positive coccidioidomycosis test results before the interview (90% vs. 59%; OR 6.1, 95% CI 2.8–13.4) and were more likely to have heard of coccidioidomycosis before their diagnosis (57% vs. 40%; OR 2.0, 95% CI 1.04–3.7). Among patients who knew about coccidioidomycosis before diagnosis, 73% first heard about it from a family member, friend, or co-worker.

## Discussion

These enhanced surveillance data provide much-needed insight into coccidioidomycosis-related illness among patients with cases reported from states where the disease is not highly endemic. Our findings are generally consistent with the similar surveillance conducted in Arizona during 2007–2008 in terms of effects on patients (*7*). Delays in diagnosis, unnecessary antibacterial use, and prolonged symptoms were common, and a high proportion of patients had culture-positive coccidioidomycosis, suggesting that less severe cases might go undiagnosed. We found several differences between patients in low- and nonendemic states, which appear to be related to the underlying populations and testing patterns.

Patients experienced considerable diagnostic delays. Although the median time from symptom onset to seeking healthcare (5.5 days) was shorter than in Arizona surveillance (11 days), the median time from seeking healthcare to diagnosis (38 days) was longer than in Arizona (23 days) and in a study of patients in Missouri (25 days from onset to diagnosis) (*7*,*10*). Compared with Arizona surveillance, the shorter time from onset to seeking care in this investigation could be related to an older patient population, whereas the longer time from seeking healthcare to diagnosis is likely related to lower coccidioidomycosis awareness in low-endemic and nonendemic states. Many patients also reported initial misdiagnosis before being tested for coccidioidomycosis; however, it is unclear whether those who said they were first diagnosed with pneumonia did, in fact, receive a correct initial coccidioidomycosis diagnosis but were either not informed of the specific etiology or did not remember being told, or whether they were truly misdiagnosed with bacterial pneumonia. Misdiagnosis seems likely because of the high proportion of patients who reported being diagnosed with another illness and receiving antibacterial medication, similar to other studies (*7*,*11*). We did not observe statistically significant differences in misdiagnosis or delays in diagnosis between low-endemic and nonendemic states, indicating a need for increased healthcare provider awareness about coccidioidomycosis in all areas.

The differences between patients from low-endemic and nonendemic states appear to reflect underlying population demographics and provider testing practices rather than differences in disease severity. Enzyme immunoassay is commonly used in highly endemic areas as an initial test for coccidioidomycosis because it is high-throughput, requires less expertise, and is more sensitive (though less specific) than other serologic methods (*2*). Providers and laboratories in low-endemic areas might be more familiar with this test than those in nonendemic areas, whereas patients in nonendemic areas were more likely to have positive coccidioidomycosis cultures. Providers in nonendemic areas might not be testing specifically for coccidioidomycosis, but rather diagnosing it incidentally, given that *Coccidioides* organisms can grow on various culture media. Nearly all cultures came from invasive procedures and <10% from sputum, suggesting that diagnosis by culture was uncommon among patients with uncomplicated primary pulmonary disease not warranting invasive procedures. Some patients from nonendemic states first sought care in endemic areas and might have had coccidioidomycosis diagnosed there; however, we did not collect data on diagnosis location, making geographic differences in testing patterns difficult to fully understand. In contrast to possible differences in providers’ knowledge of coccidioidomycosis tests, patients themselves were more likely to have known about coccidioidomycosis before being diagnosed with it and were more likely to ask for coccidioidomycosis testing if they were reported from nonendemic states. The modest awareness among these patients is probably related to the fact that a high proportion resided part-time in Arizona, where public outreach about coccidioidomycosis is frequent and awareness is likely to be greater than in other areas. Patients in nonendemic states were also more likely to know about their positive results before the interview. Possible reasons that patients did not know of their positive test results include that the patient misunderstood or did not remember their diagnosis or that the provider did not inform the patient because they believed the results were not clinically relevant. The second explanation would also support the finding that patients in low-endemic states were less likely to be prescribed antifungal medications.

The most common symptoms (cough, fatigue, and shortness of breath) and prolonged symptom duration (median 38.5 days among patients who had recovered at the time of the interview) were similar to those in Arizona patients (42 days) (*7*). Comparable to our findings, other studies show that coccidioidomycosis symptoms, particularly fatigue, can take months to resolve and profoundly impair physical activities, resulting in missed workdays and inability to perform usual daily activities (*7*,*12*,*13*). The reasons that patients in low-endemic states were more likely to report chest pain, headache, joint pain, and muscle pain than patients in nonendemic states are unclear but could further reflect geographic differences in testing practices if physicians in low-endemic areas are more likely to suspect and test for coccidioidomycosis based on those symptoms. In previous studies of coccidioidomycosis test positivity among community-acquired pneumonia (CAP) patients in highly endemic areas, myalgia (*11*) and rash (*14*) were the only clinical features that differentiated coccidioidal CAP from noncoccidioidal CAP. Another study found that chest pain was a significant predictor of being tested for coccidioidomycosis among CAP patients (*15*). In addition, approximately half of patients in our surveillance reported fever, similar to findings in Arizona surveillance (*7*). 

Potential recall bias is our investigation’s main limitation. However, patient interviews can yield insightful data about effects on patients’ daily activities and other information that might not be routinely available from medical records, such as detailed travel histories.

Travel to or part-time residence in Arizona was frequent among patients reported from nonendemic states. Approximately 40 million persons visited Arizona in 2015 (*16*), but the number of seasonal residents is more challenging to measure; the most recent figures estimated Arizona’s winter-only resident population to be ≈300,000 during 2000–2001 (*17*). These seasonal residents, also known as snowbirds, are typically retired, older adults who have higher socioeconomic statuses than others in their age group and who usually cite a more enjoyable climate as the reason for their part-time residence outside their home state (*18*). Overall, the risk for acquiring coccidioidomycosis during travel to Arizona is likely small. One expert estimated that only 1 in 17,000 visitors would experience an infection serious enough to seek medical care (*19*). However, the total number of cases estimated to occur in Arizona visitors is estimated to be ≈1,300 per year (*19*), suggesting a public health problem far larger than surveillance detects. The high proportion of cases from nonendemic states whose coccidioidomycosis was diagnosed by culture (36%, compared with <10% in Arizona surveillance) also indicates that less severe cases go undiagnosed or unreported. Obtaining a patient’s history of travel to or residence in coccidioidomycosis-endemic areas is essential for early diagnosis (*2*).

Some patients (59% from low-endemic states and 17% from nonendemic states) did not report travel to endemic areas in the 4 months before symptom onset. A study of cases in Missouri residents also found that approximately one quarter of patients did not report travel to endemic areas during the 3 weeks before symptom onset (*10*). Incomplete travel histories or travel that occurred >4 months before symptom onset likely explain the lack of recent travel to endemic areas among patients from nonendemic states in our surveillance. In low-endemic states, most cases in patients without recent travel to other endemic areas could presumably be locally acquired. A deeper understanding of the highest-risk geographic areas in those states is needed.

We classified Oregon as nonendemic; although *Coccidioides* spp. DNA was identified from several soil samples in central Oregon in 2016, the fungus has not been cultured from environmental samples (*20*). Our surveillance identified culture-confirmed coccidioidomycosis in 1 Oregon patient who did not recently travel to known endemic areas, and the acute nature of his illness did not suggest reactivation of infection acquired during his earlier travel to Mexico. The patient reported extensive exposure to alfalfa hay (of unknown source), and rare coccidioidomycosis cases have been transmitted by similar fomites (*21*), suggesting that acquisition from the local environment and remote travel are not the only possible sources of infection. Unfortunately, a suitable clinical isolate was not available for whole-genome sequencing and comparison to isolates from nearby states. Such testing, in combination with environmental isolates, has enabled identification of cases acquired from the natural environment in south-central Washington (*4*,*22*) and could allow for discovery of similar transmission in Oregon, if present.

Our results could be used to inform minor revisions to the CSTE case definition. In nonendemic areas or areas with unknown endemicity, interpretation of positive coccidioidomycosis serologic test results can be challenging if the patient is asymptomatic, has no relevant travel or an unknown travel history, or has laboratory evidence of a different disease. In this surveillance, >16% (54/339) of patients whose illness met the CSTE definition likely did not have coccidioidomycosis; all but 1 were from nonendemic states, and many had histoplasmosis, which is known to cause cross-reactions with coccidioidomycosis serologic tests (*23*). Some states are already excluding such cases from their case-counts even though the CSTE definition does not specify exclusion criteria. Similarly, the CSTE definition does not state whether cases counted in a previous year should be counted again if subsequent positive laboratory tests are reported. Most states, including Arizona, only count cases once because infection is thought to confer lifelong immunity. Seven percent of patients in our analysis self-reported a history of coccidioidomycosis, but we were not able to determine if their cases had been previously reported in other states. Last, 9% of patients we interviewed did not report symptoms, although the actual proportion could be higher because some patients were not contacted for an interview because their illnesses were thought to be clinically incompatible with coccidioidomycosis. In Arizona’s enhanced surveillance, 5% of patients had no symptoms or symptoms inconsistent with coccidioidomycosis according to the CSTE definition, suggesting that the CSTE definition’s laboratory component alone is sufficiently specific for public health surveillance (*7*). Further characterization of clinical scenarios involving asymptomatic patients with positive coccidioidomycosis tests could inform clinical practice and disease surveillance. Overall, the contributions of false-positive laboratory tests, previously reported cases, and asymptomatic cases to national-level case-counts are undoubtedly small, but they serve as examples of ways that coccidioidomycosis surveillance could be improved.

Although *Coccidioides* is most common in Arizona and California, coccidioidomycosis is a disease of national importance. Our investigation revealed many cases associated with travel to or part-time residence in highly endemic areas, as well as cases presumably acquired in Nevada, New Mexico, and Utah. Patients experienced substantial delays in diagnosis and prolonged symptoms, leading to lost productivity. The high proportion of culture-positive cases suggests that less severe cases go undiagnosed, resulting in underestimates of the actual number of cases, which is typical for public health surveillance. Greater awareness nationwide among clinicians and the public about coccidioidomycosis is needed to minimize delays in diagnosis and appropriate treatment.
